# Supplementary figures and images for: High endemicity of alveolar echinococcosis in Yili Prefecture, Xinjiang Autonomous Region, the People’s Republic of China: Infection status in different ethnic communities and in small mammals
Source: PLoS Negl Trop Dis. 2021 Jan 19;15(1):e0008891. doi: 10.1371/journal.pntd.0008891 (PMC7845998; doi:10.1371/journal.pntd.0008891)

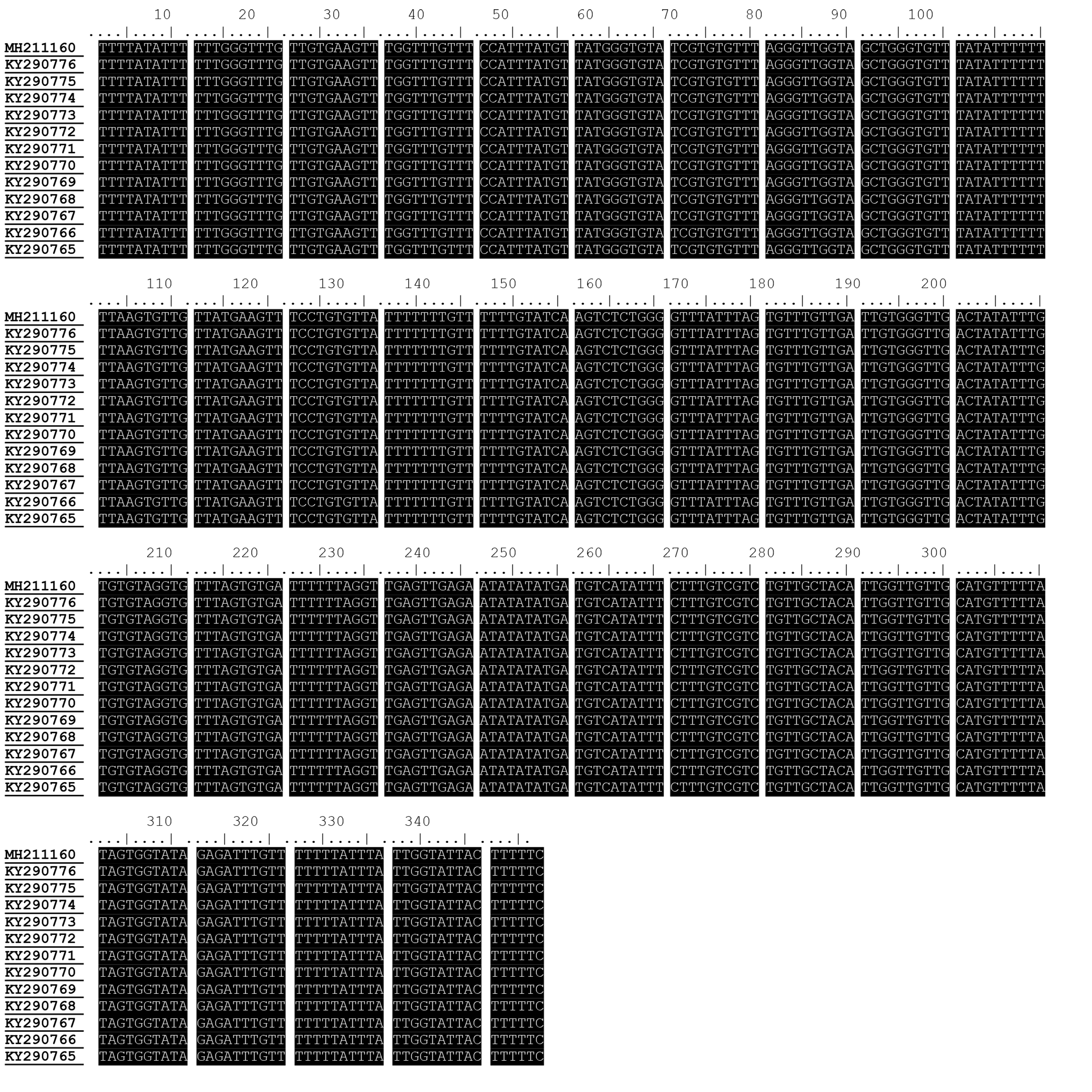

Supplement: S1 Fig — (TIF) [file pntd.0008891.s001.tif]
