# Supplementary material for: High endemicity of alveolar echinococcosis in Yili Prefecture, Xinjiang Autonomous Region, the People’s Republic of China: Infection status in different ethnic communities and in small mammals
Source: PLoS Negl Trop Dis. 2021 Jan 19;15(1):e0008891. doi: 10.1371/journal.pntd.0008891 (PMC7845998; doi:10.1371/journal.pntd.0008891)
Supplement: Attachment — Submitted filename: Response.doc [file pntd.0008891.s002.doc]

**Responses**

Questions from Editor:

Methods

-Are the objectives of the study clearly articulated with a clear testable hypothesis stated?

Response: Yes. In the study, we collected all the AE cases from the study areas in a time course of two decades. We hypothesized that: 1. AE prevalence is increasing in the 20 years period, especially in the last decade; 2, the prevalence is basically depended on the community production activities. We have changed in the introduction section.

-Is the study design appropriate to address the stated objectives?

Response: Yes. We showed that AE is increasing and may cause problem in the study area.

-Is the population clearly described and appropriate for the hypothesis being tested?

Response: We collected all the AE cases in Yili Prefecture. For addressing whether the AE infection risk is associated with people’s activity which is normally ethnic based, we searched all AE patients from whole communities from different counties and with different ethnic communities. We used the whole population as population base. We believe this is an appropriate way to test the hypotheses.

-Is the sample size sufficient to ensure adequate power to address the hypothesis being tested?

Response: We collected all the AE cases in Yili Prefecture based their location and ethical background. For addressing the upstream high-altitude areas of pasture areas, we used the whole population of 4 counties in these areas.

-Were correct statistical analysis used to support conclusions?

Response: Yes. We have cited two papers using the similar methods which support the analysis results.

10.	Zhou HX, Chai SX, Craig PS, Delattre P, Quere JP, Raoul F, et al. Epidemiology of alveolar echinococcosis in Xinjiang Uygur autonomous region, China: a preliminary analysis. Ann Trop Med Parasitol. 2000;94(7):715-729.

11.	Bresson-Hadni S, Vuitton DA, Bartholomot B, Heyd B, Godart D, Meyer JP, et al. A twenty-year history of alveolar echinococcosis: analysis of a series of 117 patients from eastern France. European Journal of Gastroenterology & Hepatology. 2000;12(3): 327-336.

-Are there concerns about ethical or regulatory requirements being met?

Response: Yes. Our study had been approved by our ethical committees.

**Questions from Reviewer #2:**

1. Information needs to be provided on when (year/season) small mammal trapping occurred.

Response: We have added “The study was carried out in the summer of 2017.” (line147-148).

2. The human aspect of the paper largely follows the methods of Zhou et al. (2000). That being said, additional information is needed in the Methods on who these AE patients represent (there is some additional information provided in the Discussion). The cases appear to be those with severe clinical disease seen in facilities with surgical capabilities. Are these the only hospitals/clinics in the region that treat AE patients? Due to the chronic nature of AE and the presumed lack of medical access for some cases, a clear description of the study population needs to be supplied so that the reader can understand the measure(s) of frequency used.

Response: Our study was an independent study including methods we used although we referred a paper published by Zhou et al. (2000). We have collected all the AE patients in this area and we used whole population as the base to calculate the prevalence. For the data of AE patients, we have discussed the prevalence of AE before 2000 showed in Zhou’s paper, which was 3.8/100,000. We showed that the prevalence of first period from 1994 to 2004 was 2.98/100,000. We have stated that those approved hospitals have expertise and facilities for surgical removing AE lesions from AE patients and are the only hospitals/clinics in the region that treat AE in this region . We have stated.

3. Even though case numbers are small, displaying the values as cumulative prevalence results in lost information. The authors are also not consistent with their frequency of infection terminology (e.g., switching between prevalence and incidence).

Response: We have changed to “prevalence”

4. Realizing the difficulty in reconnecting with patients in this location, patient follow-up information is largely incomplete. Any form of follow-up contact could only be made with 7.3% of patients (n=21) so it is difficult to draw any conclusions from this information. In addition, no information is provided about these patients’ year of treatment, age, sex, or comorbidities.

Response: We have added more information about the locations and age and sex of these followed-up patients. As the time course was covered for more than 25 years. During the period, the telephones system in China has been changed and the old phone numbers could not be connected. Only those patients(n=21) with mobile numbers from 2012 were contacted for following-up. We say “difficult” was due to the “contact phone number” lost. We have successfully contacted those 21 patients or their family members for obtaining the information, which is useful.

5. Data analysis methods are very simple and don’t make any attempt to evaluate associations between human AE cases and small mammal distribution, landscape, or farming activity as indicated by the paper’s title.

Response: We thank the reviewer’s suggestion. We have changed the title

The study is showing AE is increasing in the last 20 years and indicating that Microtus may play a role in the transmission given the high prevalence of the small mammals being infected. In term of human AE cases.

6. Does the analysis presented match the analysis plan?

Response: We believe the analysis method is a way showing the endemic situation of AE in Yili areas. We have cited two papers using the similar methods which support the analysis results.

10.	Zhou HX, Chai SX, Craig PS, Delattre P, Quere JP, Raoul F, et al. Epidemiology of alveolar echinococcosis in Xinjiang Uygur autonomous region, China: a preliminary analysis. Ann Trop Med Parasitol. 2000;94(7):715-729.

11.	Bresson-Hadni S, Vuitton DA, Bartholomot B, Heyd B, Godart D, Meyer JP, et al. A twenty-year history of alveolar echinococcosis: analysis of a series of 117 patients from eastern France. European Journal of Gastroenterology & Hepatology. 2000;12(3): 327-336.

7. Are the results clearly and completely presented?

Response: We have shown the endemic situation of AE in the study area. We have added more tables showing the data.

8. Are the figures (Tables, Images) of sufficient quality for clarity?

Response: We have added tables for showing more data and changed the tables and images.

**Questions for Results**

Much of the results can be summarized in tables rather than presented in the text.

Response: Based on the reviewer’s suggestions, we have added tables showing more data and shorted the sentences in the text.

The abstract states that "...E. multilocularis DNA sequences from small mammals were identical to isolates from local AE cases." However, I don't see where sequence data from local human cases were obtained or discussed as part of this study. Please clarify.

Response: We are sorry for that. We did the sequence analysis of E. multilocularis sequences from human cases in our previous study. We have added a table for showing these sequences and the sequence references.

Please confirm the geographic origins of sequences KT965441 and KT965439. They differ between the text and figure 4.

Response: We have confirmed the sequences accession numbers and changed.

**Conclusions**

-Are the conclusions supported by the data presented?

Response: Yes. .

-Are the limitations of analysis clearly described?

Response: The limitation of the study is that no data showing the dog infection status with adult worms of E. multilocularis due to the bio-safe management in our hospital. However, the family of sheep-farmers are normally raising one or more sheepdogs. These dogs play an important role in transmission of AE to humans. We have added the statement into the discussion.

-Do the authors discuss how these data can be helpful to advance our understanding of the topic under study?

Response: In the study, we identified that high altitude areas had higher prevalence in both human communities and small mammals then these low altitude areas. Our data show that the prevalence of AE is likely depended on the landscape, especially the altitude in the mountain areas and species of small mammals. In the investigation areas, a species of small mammals-Microtus voles play an important role in the transmission of AE. In addition, our findings suggest that increased testing of these specific communities could identify more cases for treatment, resulting in more people receiving the treatment they need. Emphasise that the findings will provide new information that could improve the targeting of disease control efforts, making them more effective. We have changed in the discussion.

**Results in the Discussion**

Reviewer #2: There is quite a bit of repetition of Results in the Discussion.

Reponse: We have updated the text in response to the comment raised.

It would be helpful if the flow of the Discussion mirrored the Methods and Results rather than moving from human AE frequency, to small mammal distribution, back to clinical management of AE cases.

Response: We have changed and mirrored Results in the discussion section.

Reviewer #2: The authors refer to the use of experimental animals (line 100). Please clarify if this is simply another reference to the trapped small mammals or if there was a separate group of animal subjects.

Response: This was a reference to the trapped wild small mammals. We have updated the text to make this clearer.

--------------------

Summary and General Comments

Reviewer #1:

**Major points**

1. Line 132 & 200: "To calculate the prevalence of AE" and "to estimate the annual prevalence over the two decades". As the authors investigated the occurrence of new AE cases during a period of time, it is more accurate to use "Incidence".

Response: We have changed to “prevalence”. We have cited to Bresson-Hadni’ paper and think CP is more suitable.

2. Line 168-176: No need to provide much details on DNA extraction method, "according to the manufacturer’s instructions" is fine.

Response: We have deleted.

3. No data have been provided for sex distribution of the patients. Please tabulate data on the age- and sex-specific incidence of AE in different ethnicities/regions.

Response: We have added the data in two tables, Table 4 and Table 3.

4. Please provide the Mean and Median age of the patients.

Response: We have used both Mean and Median (41.86/46) in text. (Line 236-237)

5. Patient survival data are very valuable. It would be great to tabulate survival data and provide 5-year survival rates (absolute or relative) of AE patients (the percentage of AE patients living five years after the disease is diagnosed).

Response: We have showed 21 patients who were contactable. However, all those patients had their surgery less than 5 years (from 2012-2016), it is difficult for us to calculate 5-year survival rates.

6. Also please report the Median survival that is the period of time after which 50% of the patients have died and 50% have survived.

Response: For these 6 AE patients without surgical removal of AE lesions and taking albendazole, the median survival time was 3.5 years. We have changed. (Line 262-265)

7. Were there any patients undergoing liver transplantation? If yes please provide details of the prognosis and patients conditions.

Response: None of these patients received liver transplantation.

8. The method used for detecting protoscoleces in the cysts is not clear.

Response: For Microtus voles, we sectioned the liver tissues and used H&E staining. For AE patients, we did not check protoscoleces. We have mentioned in the text in M&M in page6.

9. Line 268: "We also “captured” 16 from HAPA in Chabuchaer County", this county has already been categorized in LAAA region! Please clarify.

Response: Only one very small area is close to HAPA and very few Microtus spp were collected. We have deleted the statement.

10. Please provide chi-square analysis results for comparing differences among time periods and ethnic groups.

Response: The study involves the number of patients in each county, ethnic group and gender. It is not a sample survey, so there is no need to do chi-square test.

11. Contradictory statements written in line 280-283 on Rhombomys and Meriones infection!

Response: We are very sorry for the wrong statement. We have replaced the sentence with “No *Mus musculus* (n=232) and *Rattus norvegicus* (n=29), *Cricetulus migratorius* (n=6) and *Meriones tamariscinus* (n=6) (Table 5) were infected with *E. multilocularis* metacestodes.”(Line 293-295)

12. In Fig. 1 no sampling site (cross) has been specified for the farming areas. Does it mean all the samples were taken from the pasture areas?

Response: We have added the site in Figure 1.

13. According to the Fig. 2, several AE cases were observed in adolescents under 15 years of age. this is an interesting finding. Please provide additional data on these AE patients. The nature of AE in children and adolescents is unknown therefore these type of information are very valuable.

Response: Thanks very much for the suggestion. Four young patients with one at age 12 (Hui) and three (Kazaks) at age 15. All of them were from sheep-farmer families in the high altitude counties. We have added sentences in the text.(line 241)

14. It is not clear which haplotype from where? has the highest frequency. To understand the extent of genetic variation within *E. multilocularis* isolates from Xinjiang please provide (in a table) more data on the frequency distribution of 16 haplotypes according to different locations and other variables if applicable.

Response: We have added a Table (Table 6) indicating the haplotypes of *E. multilocularis* in Yili Prefecture.

15. The study found that 73.08% of AE patients had been diagnosed in the last 10 years, please specify that if this is because of more diagnostic facilities provided in recent years or more active case-finding or more intense disease transmission or more patients seeking treatment etc? please elaborate more on this.

Response: In 2013, a survey for 4000 residents was performed in each of the eight counties and there were more AE cases were identified in this year. We have added more discussion.

“An ultrasound survey about 4000 residents per county on echinococcosis was preformed in 2013(data not shown). In this year, a total of 33 AE cases were registered, which is 11.40 more than the average (21.60) of AE cases from 2012 to 2016 (with AE cases being 20, 33, 20, 18 and 17 respectively), indicating that the mass screen had a limit impact on the growth of AE cases in the last 10 years.”

16. "Microtus suppress the production of PSCs." As the method used for PSC detection is not clear, this statement is not justifiable. Several studies across the continents have shown that different *Microtus spp*. are highly potent intermediate hosts for E. multilocularis, i.e they are favorite preys for the definitive hosts, susceptible to *E. multilocularis* with a high population density (see Ecology and Life Cycle Patterns of Echinococcus Species, Adv. Parasitol. 2017; and Giraudoux et al. 2013). Therefore as no evidence has been presented in the study, it is quite premature to say "*Microtus spp*. suppress protoscoleces production". In addition the authors demonstrate further contradictions in the Title (line 1-3) and Conclusion section (line 414-415) when they talked about the crucial role of *Microtus* in AE transmission in the region ! Please elaborate more on this.

Response: We have shown that Microtus had a relatively high prevalence for infecting AE. They likely play an important role in the transmission of AE. We have deleted line 414-415. We have discussed.

17. Please provide separate descriptions for the two phylogenetic trees in the legend for Fig. 4.

Response: We have carefully changed. It is divided into Figure 4a and Figure 4b.
Fig 4a :“We amplified 43 copies of gene cox1 and divided them into 16 haploids (Fig 4a), with XH1 at the most and 21 samples working”(Line306-310)

**Minor points**

1. line 177: PCR or nested-PCR ?

Response: It is nested-PCR, we have carefully modified.

2.Line 206: "Kazak (53.50% (153/286))" is redundant.

Response: We have deleted.

3. line 280: "2 of 25 Rhombomys opimus," is redundant.

Response: We have deleted.

4. Line 297: the GenBank accession number KT965411 is for Influenza A virus !

Response: It is KT965441, we have carefully modified.

5. Please be consistent with reporting decimals, choose either one or two decimal places all over the manuscript.

Response: We have carefully modified, such as in the first paragraph of the results:

“The first AE case in our data set was identified in 1989 in Yili Friendship Hospital. By 2016, a total of 286 cases were diagnosed and registered in Yili Prefecture（Table 2）. From 2005 to 2015, the CP was 7.77/100,000 (209 cases/2,690,960 residents (2015)), accounting for an average annual prevalence (AP) of 0.70/100,000, which is 2.59 times than the AP(0.27/100,000) in the first period from 1994 to 2004. We found 73.08% (209/286) of patients had been diagnosed from 2005 to 2015 (Fig 2a). Of these AE patients, 153 cases were Kazak accounting for 53.50% of the total AE cases, followed by Han (18.88% (54/286)), Uygur (12.24% (35/286)), Hui/DongXiang (8.39% (24/286)), and Mongol (6.29% (18/286))”.

Also, we have changed in the all tables.

6. line 365: Em > *E. multilocularis*

Response: It is *Em*, we have carefully modified.
