# Supplementary material for: High endemicity of alveolar echinococcosis in Yili Prefecture, Xinjiang Autonomous Region, the People’s Republic of China: Infection status in different ethnic communities and in small mammals
Source: PLoS Negl Trop Dis. 2021 Jan 19;15(1):e0008891. doi: 10.1371/journal.pntd.0008891 (PMC7845998; doi:10.1371/journal.pntd.0008891)
Supplement: Attachment — Submitted filename: Response.docx [file pntd.0008891.s003.docx]

Comments

Reviewer #1:

- Ref to the Reviewer #2 comment, in Discussion, the "Treatment and follow-up" section has come after small mammal section, please follow the reviewer comment and put all clinical human data together.
Response: We have changed and removed the related discussion.

- Line 168: "RNA free-water" is not correct.

Response: We have changed this to" RNase free-water "

- "there is no need to do chi-square test" So please modify the Statistical analysis section in M&M (Ref to the Reviewer #1 comment).

Response: This is a wrong statement. We have changed. The Chi-Square test was used to compare the differences in prevalence between time periods, counties, and ethnic groups. We have stated this in the Materials and Methods section, and have presented these results throughout the results section.

- Ref to the Reviewer #1 comment, please note that Em, Eg, Ro, As, Et are taxonomically / biologically non-sense. Please use standard scientific nomenclature.

Response: We have revised and used the standard scientific nomenclature.

- English writing needs a revision.

Response: We have fully edited the MS including English.
- line 442: was preformed > was performed

Response: “preformed” has been changed to “performed”.

Reviewer #2: The manuscript is somewhat improved. However, numerous grammatical errors still exist. In addition, the authors make several statements regarding relationships between human AE cases and perceived risk factors that are not directly supported by study data. Please find my specific comments below (line numbers refer to the Word document with track changes).

Response: We have extensively edited the MS including English. CM and DPM have carefully checked the whole MS.

Based on the study design, I would suggest that the authors moderate the statement, “The overall results indicate that the transmission of AE is highly associated with landscape and distribution of *M. obscures* in Yili Prefecture” since they didn’t evaluate landscape or statistical associations between small mammal distribution and human AE cases. (lines 50-52) [Wording in the author summary is slightly better.]

Response: We have changed to:

“The overall results show that Yili Prefecture is a highly endemic area for AE and that the high-altitude pasture areas favorable for *M. obscurus* may play an important role in its transmission in this region.”

I’m also not sure how the authors came to the conclusion that “…sheep-farming activity is a risk for infection”. (line 43).

Response: We have discussed and changed in the conclusion section as follows:

**Conclusion**

In summary, Yili Prefecture is a high endemic area for AE and *Microtus* *spp* voles likely play a crucial role in its transmission. The high prevalence of AE in the Kazak and Mongolian communities is probably due to farmers raising sheep in the summer season in high-altitude pasture areas where there are significant numbers of small mammals infected with *E. multilocularis;* this enhances AE transmission due to close contact of these farmers with their sheep dogs.

Information in Table 6 (*E. multilocularis* haplotypes and accession numbers) needs to be elaborated upon. It is not clear if these samples were from small mammals or humans (or if these are samples from the current study or a previous study). The authors also need to provide support for the statement, “…*E. multilocularis* DNA sequences from small mammals were identical to isolates from local human AE cases”. (lines 49-50)

Response: All listed sequences were from small mammals and from the present study. We have updated the table caption to state this “Table 6. *E. multilocularis* haplotypes characterized by partial cox1 sequences used for phylogenetic analysis. All samples were from small mammal hosts collected during the current study.” One of the Nad2 sequences is identical to the sequence from human cases in our previous study. We have shown

“The another one predominant (60/97) *E. multilocularis* Nad2 haplotypes (MH211160) from small mammals was identical to the sequence isolated from local human AE cases in our previous study [13] (Supplementary Figure1). ”

The statement in lines 93-96 (“In this study, we retrospectively collected human AE cases from eight counties and one city in Yili Prefecture, which shows that AE prevalence was increasing in the 20 years period, especially in the last decade and the prevalence of AE is likely associated with landscape features.”) doesn’t belong in the introduction. Again, this study did not evaluate landscape features.

Response: We have moved the sentence from the introduction section．

We have changed in the results and discussion sections.

Such as, in the Discussion section, (page #, line ##: we stated:”……the prevalence of AE is likely associated with altitude associated landscape features such as pasture areas.”) (lines 335-339)

We found that there were only two species of small mammals from the HAPA, and seven species from the LAAA. The results indicate that the prevalence of AE was higher in areas of relatively low small mammal biodiversity than in more diverse host communities. In low diversity small mammal communities, potential intermediate host species prone to population outbreaks. Also, grass productivity and forest cover are correlated with rainfall and temperature, which are correlated (locally) with altitude (29). In brief, the notion that landscape, small mammal host biodiversity and their population dynamics may protect humans from AE transmission where they prevent population outbreaks of a few specific small mammal host species, but altitude is the opposite of small mammal host biodiversity and their population dynamics. (lines 459-469)

The authors state that, “The Chi-Square test was used to compare the differences in prevalence between time periods, counties, and ethnic groups.” However, I don’t see where chi-square values (and associated p-values) for time periods are presented in the Results section. In addition, chi-square findings for ethnic groups and counties are not clearly presented (please also see my comment regarding the figure 2 legend). (lines 199-200)

Response: We have changed. The Chi-Square test has been presented at appropriate positions throughout the results section (rather than as a stand-alone table). (lines 196-200)

Are the provided ages for the follow-up patients, their ages at the time of follow-up or at diagnosis? What about those who had died during the follow-up period? In general, the “follow-up of AE patients” section is very difficult to follow and would benefit from restructuring and clarification when presenting information about time since diagnosis and time since surgery for both patients who are still alive and for those who have died.

Response: For human AE cases, we retrospectively reviewed all the registration data. Follow-up was done recently. For better understanding, we have revised the statement.

Are the provided ages for the follow-up patients, their ages at the time of follow-up or at diagnosis?

Response: ages provided were the ages at the time of contacting with. The years for survival is the years after being diagnosed. We have stated this in the revision.

What about those who had died during the follow-up period?

Response: we only showed 21 of these AE cases being contacted with. The survival time is the time since diagnosis.

Please clarify what is meant by “21 samples working”. (line 343)

Response: Sorry, this is a typo mistake. We have changed this from “21 samples working” to “21 samples”.

Figure 2 legend- What do the authors mean by “an asterisk indicates above average”?

Response: “an asterisk indicates above average” means that these figures are significantly higher than the average of prevalence in the whole areas. We have noted.

Figure 3 doesn’t appear to be referenced in the text.

Response: Figure 3 has been referenced in the text – “Four *Ellobius tancrei* were captured with two (2/4) infected with metacestodes, both with PSCs of *E. multilocularis* (Fig 3).”(line 328-330)

The authors added the following section to the revised document: “An ultrasound survey about 4000 residents per county on echinococcosis was preformed in 2013(data not shown). In this year, a total of 33 AE cases were registered, which is 11.40 more than the average (21.60) of AE cases from 2012 to 2016 (with AE cases being 20, 33, 20, 18 and 17 respectively), indicating that the mass screen had a limit impact on the growth of AE cases in the last 10 years.” This statement is confusing and I don’t think it addresses the question regarding possible changes to patient access to care over the study time period.

Response: We have changed to the following statement in the discussion section.

“In the last 10 years, the extensive education program and improvement of diagnosis ability may impact the growing of AE. However, the data from the last 5 years showed that 20, 33(a partial mass Ultrasounography was done in 2013), 20, 18 and 17 respectively were identified from 2012 to 2016, indicating a trend of decreasing of AE cases.”

Overall, the flow of the Discussion remains disjointed.

Response: We have revised the discussion making numerous changes to improve the flow of this section.
