# Supplementary material for: High endemicity of alveolar echinococcosis in Yili Prefecture, Xinjiang Autonomous Region, the People’s Republic of China: Infection status in different ethnic communities and in small mammals
Source: PLoS Negl Trop Dis. 2021 Jan 19;15(1):e0008891. doi: 10.1371/journal.pntd.0008891 (PMC7845998; doi:10.1371/journal.pntd.0008891)
Supplement: Attachment — Submitted filename: Dear Editor-response.doc [file pntd.0008891.s004.doc]

**Response**

Comments

In the author summary the way prevalence is reported is just wrong. Prevalence is the proportion with the disease at one point in time...ie if an ultrasound study found 31 cases in as survey of 100,000 the prevalence is 31/100,000 = 0.000031 or 0.0031%. If they are reporting incidence it should be the (average) annual incidence, which if it is 31.62 cases/100,000 in 27 years (as implied by the text) then the mean annual incidence is 1.17 cases per 100,000. Since the cases were based on hospital records of patients (as described in materials and methods) presenting for treatment, then the data is almost certainly incidence data and hence should be reported as an annual incidence of  0.49-1.17 cases / 100,000, Thus the annual incidence of 0.49-1.17 cases/100,000 in the high altitude mountainous areas was higher than the annual incidence of 0.180-0.282 cases per 100,000 seen in the low level areas. Likewise Mongolian (annual incidence 1.987/100,000) and Kazakh (annual incidence
0.897/100,000) had a higher incidence…...

Response: Thanks very much for the professional suggestions. We have changed the “prevalence” to “incidence” for its use in description of human infections. In fact, in the article we compared the incidence of human AE cases in two periods, from 1994 to 2004(before echinococcosis control program commence in 2005), 11 years and from 2005 to 2015, the second 11 years(after echinococcosis control program commence). We have discussed in the discussion section. We have used “cumulative incidence” replaced by “cumulative prevalence”. The original draft indicated 2016 in somewhere of the text as we collected some cases in 6 months of 2016. In the MS, we removed all the cases of 2016. So, the end of year for collecting data is 2015. We have changed. The details of the changes are as the followings:

Page 1, line2:….. the prevalence” changed to “ …… the infection situation……”.

Page 2, line39, 42: “prevalence (CP)” changed to “higher cumulative incidence (CI”) and we have changed CP to CI in the rest of the text.

Page 2, line 42: “prevalence” changed to “incidence ”

And also include : page 3, lines 63 and 66; page 4, line 100; page 5, line 139; page 7, line 196; page 7, line 200, line 203, line 205; page 9, line 233; page 10, line 244; page 13, line 348, line 355, line 358; page 16, line 379, line 384; page 17, line 387; page 20, line 477;

In the results section they have reported “annual prevalence” in some cases. This is not prevalence data, it is annual incidence, again correct the terminology. Likewise it is trend in incidence NOT prevalence. They have no prevalence data in humans in this study. They have correctly reported prevalence data in small mammals (ie proportion of small mammals infected).

Response: “prevalence” has been changed to “incidence”.

Reviewer #2: Please clarify if human data were collected from 1989-2015 or 1989-2016. The end date varies throughout the abstract and text.

Response: We collected the human cases from 1989 to 2015. But the compared the cumulative incidence of two periods: from 1994 to 2004 and from 2005 to 2015, with each period of 11 years. We have indicated in the text.

Table 5- change infection rate to infection prevalence.

Response: We have changed to “prevalence”.
