# Supplementary material for: High endemicity of alveolar echinococcosis in Yili Prefecture, Xinjiang Autonomous Region, the People’s Republic of China: Infection status in different ethnic communities and in small mammals
Source: PLoS Negl Trop Dis. 2021 Jan 19;15(1):e0008891. doi: 10.1371/journal.pntd.0008891 (PMC7845998; doi:10.1371/journal.pntd.0008891)
Supplement: Attachment — Submitted filename: Response.docx [file pntd.0008891.s005.docx]

**Response**

There has been a modest attempt in the results section to include the data as annual incidence per 100,000, but this has not been included in the abstract and summary.  Assuming this represents the number of cases over the26 years of the study period, then the mean annual incidence is 2.1 cases per 100,000 per year, which is typical of incidence in highly endemic regions. Also, since there were only 286 cases reported in this period from the relevant counties in Yili prefecture

over 26 years. This is not a problem, but then converting it to cumulative incidence in the manner the authors have, give a false impression as to the extent of the disease. This manuscript should not be published until they have standardized the incidence data as cases per 100,000 per year throughout the manuscript and removed the emphasis on cumulative incidence.

***Response:*** We have changed all Cumulative incidence (CI) into annual incidence throughout the MS and removed the CI in both result and discussion section
